# Detection of Abnormal Changes on the Dorsal Tongue Surface Using Deep Learning

**DOI:** 10.3390/medicina59071293

**Published:** 2023-07-13

**Authors:** Ho-Jun Song, Yeong-Joon Park, Hie-Yong Jeong, Byung-Gook Kim, Jae-Hyung Kim, Yeong-Gwan Im

**Affiliations:** 1 Department of Dental Materials, Dental Science Research Institute, School of Dentistry, Chonnam National University, Gwangju 61186, Republic of Korea; songhj@jnu.ac.kr (H.-J.S.);; 2 Department of Artificial Intelligence Convergence, Chonnam National University, Gwangju 61186, Republic of Korea; 3 Department of Oral Medicine, Dental Science Research Institute, School of Dentistry, Chonnam National University, Gwangju 61186, Republic of Korea

**Keywords:** artificial intelligence, deep learning, dorsal tongue, oral mucosa, tongue, tongue diseases, tongue mucosa

## Abstract

*Background and Objective*: The tongue mucosa often changes due to various local and systemic diseases or conditions. This study aimed to investigate whether deep learning can help detect abnormal regions on the dorsal tongue surface in patients and healthy adults. *Materials and Methods*: The study collected 175 clinical photographic images of the dorsal tongue surface, which were divided into 7782 cropped images classified into normal, abnormal, and non-tongue regions and trained using the VGG16 deep learning model. The 80 photographic images of the entire dorsal tongue surface were used for the segmentation of abnormal regions using point mapping segmentation. *Results*: The F1-scores of the abnormal and normal classes were 0.960 (precision: 0.935, recall: 0.986) and 0.968 (precision: 0.987, recall: 0.950), respectively, in the prediction of the VGG16 model. As a result of evaluation using point mapping segmentation, the average F1-scores were 0.727 (precision: 0.717, recall: 0.737) and 0.645 (precision: 0.650, recall: 0.641), the average intersection of union was 0.695 and 0.590, and the average precision was 0.940 and 0.890, respectively, for abnormal and normal classes. *Conclusions*: The deep learning algorithm used in this study can accurately determine abnormal areas on the dorsal tongue surface, which can assist in diagnosing specific diseases or conditions of the tongue mucosa.

## 1. Introduction

The tongue is a free-moving structure of the oral cavity that consists mainly of muscles, and its surface is lined by the oral mucosa. In addition, the dorsal surface of the tongue is covered with specialized mucosa containing papillary structures. In general, there are four different forms of lingual papillae on the tongue surface: filiform, fungiform, circumvallate, and foliate papillae. The distribution of the different lingual papillae is the basis of the visual patterns on the dorsal surface of the tongue. Various local and systemic diseases or conditions affect the oral mucosa of the tongue. When the tongue mucosa undergoes pathological changes due to diseases and conditions, its appearance is altered. In addition, some changes are specific to particular diseases and can provide crucial information for diagnosis.

A fissured tongue is a common condition characterized by multiple grooves or furrows of 2 mm to 6 mm depth on the dorsal tongue surface. Geographic tongue is another common benign condition affecting the tongue, characterized by erythematous, well-demarcated areas on the tongue mucosa due to atrophy of the filiform papillae. Several conditions or diseases cause an atrophic tongue. Diffuse erythema with a smooth, atrophic dorsal surface typically represents the erythematous type of oral candidiasis. Nutrient deficiencies such as vitamin B12, folic acid, or iron deficiency cause glossitis and anemia, resulting in atrophy of the papillae on the dorsal tongue surface [1,2]. An atrophic tongue has also been reported to be associated with Sjögren syndrome [3]. Autoimmune bullous diseases such as pemphigus vulgaris, paraneoplastic pemphigus, and mucous membrane pemphigoid cause irregularly shaped erythema, erosion ulcers, and blistering of the tongue mucosa [4]. When the tongue mucosa is affected by the reticular form of oral lichen planus, the papillae on the tongue surface are replaced by smooth, whitish plaques. Hypertrophy of the fungiform papillae has been reported to be associated with diseases such as guttate psoriasis [5] or medications such as cyclosporin A [6]. In patients with a coated tongue, the tongue’s dorsal surface appears whitish and slightly thickened due to the accumulation of desquamated epithelial cells and numerous bacteria. A “hairy tongue” is a condition in which the filiform papillae are elongated and hair-like, cover the dorsal surface of the tongue, and often appear yellow, brown, or black due to staining.

In medical practice, clinicians try to detect morphological abnormalities suggestive of disease based on visual examination. Clinical diagnostic tools, including toluidine blue dye, OralCDx brush biopsy, salivary diagnostics, and optical imaging techniques such as chemiluminescence imaging, auto-fluorescence imaging, narrow-band imaging, reflectance confocal microscopy, and optical coherence tomography, have been used for the early detection of oral potentially malignant disorders [7,8]. Recently, deep learning (DL) methods have been applied to improve the diagnosis of oral diseases using classifications [9,10,11,12], object detection [13,14,15], or semantic segmentation [16,17] based on dental image data. DL techniques offer promise in enhancing early detection and improving decision-making for oral squamous cell carcinoma patients through automated image analysis, facilitating cancer detection, classification, segmentation, synthesis, and treatment planning [18]. Tongue squamous cell carcinoma patients at an advanced stage could be stratified into different likelihoods of overall survival using a machine learning model based on an ensemble machine learning paradigm, helping guide evidence-based treatment decisions [19]. DL models using oral endoscopic images were utilized to identify patients with tongue cancer, achieving acceptable performance in diagnosis. The best model, DenseNet169, exhibited comparable performance to general physicians and oncology specialists in sensitivity, specificity, and accuracy [20]. DL models demonstrated high accuracy in diagnosing gastric cancer based on tongue images and the tongue coating microbiome, suggesting that tongue images can be a reliable and superior method to outperform conventional blood biomarkers [21].

DL can help segment abnormal regions on the surface of the patient’s tongue, regardless of their shape or area. Although some studies have reported predicting lesions using the DL method from oral photographic images [22,23], there are no studies segmenting abnormal regions on tongue images. Semantic segmentation or U-Net DL methods could be considered to segment abnormal and normal regions on the tongue surface accurately. However, a sufficient amount of data must be prepared to effectively employ these methods. Furthermore, due to the mixture of various types of abnormal areas with normal regions, it is necessary to enlarge the images for precise evaluation during the labeling process. Consequently, direct segmentation using conventional DL methods becomes challenging. In this study, we employed the point mapping method to illustrate the distribution of abnormal regions. This method involves dividing the tongue image into numerous points and predicting the class for each point. Subsequently, different colors are assigned to represent the abnormal, normal, and other regions. The objective of this study was to investigate the efficacy of DL in detecting abnormal areas on the dorsal tongue surface in both patients and healthy adults. These findings hold the potential to contribute to the diagnosis of tongue mucosa-related diseases significantly.

## 2. Materials and Methods

### 2.1. Collection of Clinical Photographic Images

Ethical approval was obtained from the Institutional Review Board of the Chonnam National University Dental Hospital (CNUDH-2022-008). We have read the Helsinki Declaration and have followed the guidelines in this investigation. The 175 clinical photographs of the tongue affected by various diseases and conditions were collected from the picture archive from January 2013 to January 2021 in the Department of Oral Medicine, Chonnam National University Dental Hospital. The picture archive for clinical photographs taken of patients’ oral and maxillofacial lesions is a hard disk storage space on the personal computer in the Department of Oral Medicine. The selected patients were 19 years of age or older, and the selected photographs included the tongue’s partial or entire dorsal surface.

Clinical photographs of the dorsal tongue surface with a normal appearance were obtained from asymptomatic, healthy adults aged between 19 and 39 years. We excluded subjects with oral mucosal diseases or a history of systemic disease that may cause oral mucosal changes from their medical history and clinical examination. Thirty-nine subjects (22 women and 17 men, with a mean age of 27.0 ± 2.5 years) finally enrolled after a screening evaluation among those who volunteered for the study. Each subject was informed about the study procedure, and written consent was obtained before participation in the study. The subject lay dorsally on a dental chair with the head supported. Subjects were asked to push their tongue forward, and clinical photographs were taken with a digital camera showing the partial or entire dorsal surface of the tongue.

The digital camera set used in clinical photography consisted of the following elements: a Nikon D80 body, an AF Micro Nikkor 105 mm 1:2.8 D lens, and a 4-fiber flashlight diffuser. The digital SLR Camera Nikon D80 has a 23.6 × 15.8 mm RGB CCD image sensor with effective pixels of 10.2 million. Photograph files taken with the Nikon D80 had the following properties: an image size of 3872 × 2592 pixels, 300 DPI resolution, 24 bits, sRGB color, and the JPG file type.

### 2.2. Preprocessing of Photographic Images for Deep Learning

**Figure 1:**
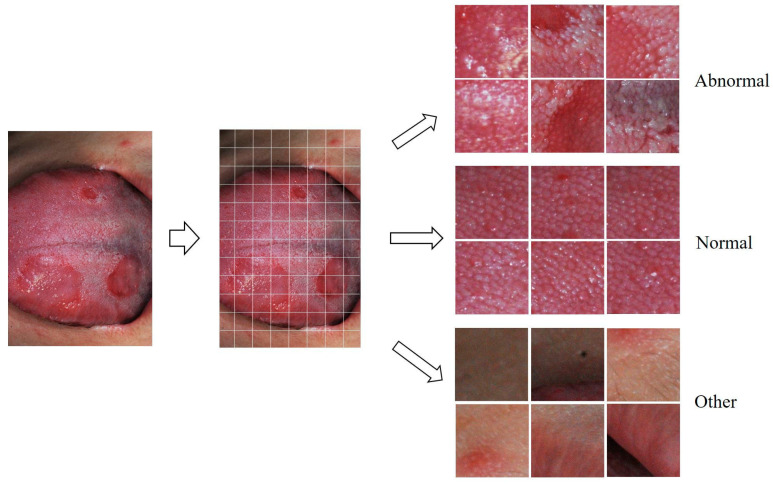
Photographs of the dorsal tongue surface were divided into small square regions, and each crop image was classified into ‘normal,’ ‘abnormal,’ and ‘other.’.

In the photographic images of the tongue collected in this study, some images showed the normal or abnormal condition on the entire tongue surface, while others showed that the normal and abnormal regions were all mixed in one image. In addition, the photographic magnification was different, such as a photograph showing the entire tongue and a locally enlarged photograph. The original photographs of the dorsal tongue surface were divided into 48 to 180 small square regions to have a similar density of lingual papillae in each image. Forty-eight (6 × 8) crop images were obtained from the photographs of parts of the tongue taken at the maximum magnification of the lens. In contrast, 180 (12 × 15) crop images were obtained from photographs taken to include the entire dorsal surface of the tongue at reduced magnification. For example, in Figure 1, since the tongue photo image with a resolution of 2592 × 3872 pixels in width × height was divided into 8 × 12 pieces, one small crop image had a resolution of width × height = 324 × 323 pixels. The data set for deep learning training was composed of these cropped images.

**Figure 2:**
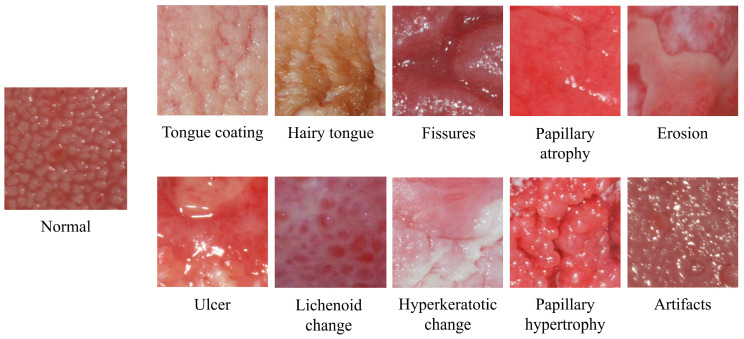
Examples of crop images showing normal and abnormal or pathological changes of the dorsal tongue surface.

Each crop image was assigned to ‘abnormal,’ ‘normal,’ and ‘other’ classes (Figure 1). A specialist in oral medicine with 24 years of experience labeled these crop images. The cropped images from patients showing normal features were excluded from those with abnormal or pathologic changes. Conversely, crop images showing abnormal features were excluded from those from asymptomatic healthy subjects. The criteria for determining abnormal or pathological changes in crop images are shown in Table 1, and examples of each are shown in Figure 2.

### 2.3. Classification of Crop Images Using the VGG16 Model

The VGG16 (Visual Geometry Group) [24], ResNet-50 (Residual Network) [25], and Xeption (Extreme Inception) [26] CNN DL models were used to classify the constructed dataset. The VGG16 model has a simple and uniform architecture consisting of 16 weighted layers and small convolutional filters (3 × 3) that help to capture fine-grained features [24]. The ResNet50 model, based on the concept of residual learning, has a 50-layer convolutional neural network and can overcome the vanishing gradient problem [25]. Xception, based on the Inception architecture, achieves efficiency by using extreme separable convolutions [26].

Of the crop images, 68.8% were used as training data, 15.4% as validation data, and 15.8% as test data (Table 2). All crop images were resized to 96 × 96 pixels, and image augmentation was performed by horizontal flip, vertical flip, and rotation of each image by 90°. To avoid overfitting, a dropout (0.5) layer was applied after the fully convolutional network layer. All layers of the models were fully trained. The stochastic gradient descent (learning rate = 0.005, momentum = 0.9) was used as the optimizer. The categorical cross-entropy method was applied as the loss function. The total number of epochs was 50.

The 1229 test images were predicted using trained weight values, and a confusion matrix was obtained (Table 3). The number of true positives (TP), false positives (FP), false negatives (FN), and true negatives (TN) were counted for each class, and the precision, the recall, the F1-score (harmonic mean of precision and recall), and the accuracy were calculated as follows:

$$\mathrm{Precision}=\frac{TP}{TP+FP}\;\mathrm{Recall}=\frac{TP}{TP+FN}\;F1\;\mathrm{Score}=2\times \frac{\mathrm{precision}\;\times \;\mathrm{recall}}{\mathrm{precision}\;+\;\mathrm{recall}}\;\mathrm{Accuracy}=\frac{TP+TN}{TP+TN+FP+FN}$$

In addition, after drawing the receiver operating characteristic (ROC) curve for each group, the area under the curve (AUC) value was calculated.

### 2.4. Point Mapping on the Dorsal Tongue Surface

**Figure 3:**
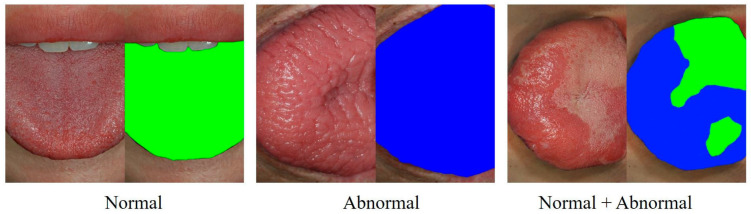
Examples of labeling abnormal and normal regions of the dorsal tongue surface with green and blue colors.

The original photographs showing the dorsal surface of the entire tongue were mapped using points to indicate normal and abnormal regions by the following method: Photographs of the entire dorsal tongue surface of 80 patients who had not participated in previous VGG16 training were prepared. Each of them was labeled ‘abnormal,’ ‘normal,’ and ‘other’ regions using blue and green colors, as shown in Figure 3. The oral medicine specialist evaluated the images at various resolutions by freely enlarging or reducing the original photographic images in a graphics editing program (PaintShop Pro, version 9.01, Jasc Software, Eden Prairie, MN, USA). Detailed papillary structures were evaluated within the magnification range as well as the overall papillary pattern of the dorsal tongue surface. Regions on the dorsal tongue surface with normal mucosa were labeled in green, and those showing abnormal or pathologic changes were labeled in blue (Figure 3).

**Figure 4:**
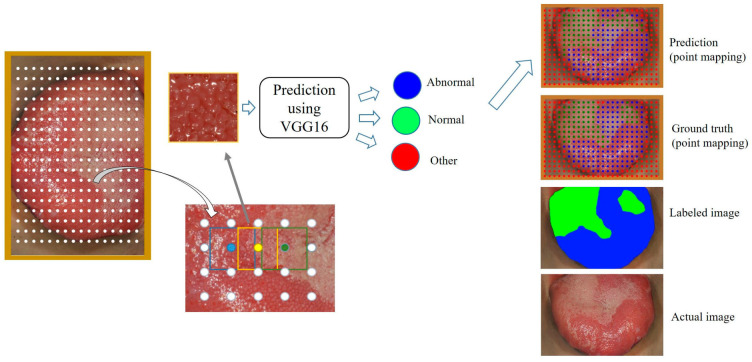
Small-size images were cropped from the original photographic images of the entire dorsal tongue surface. These small images were predicted to be abnormal, normal, or other.

In order to apply the previously learned DL result for the classification of crop images, the original photographic images were divided into small-size images similar in size to the previously trained images. The boundaries of these images were partially overlapped to increase the area’s density to be analyzed. Three hundred eighty-four images (16 × 24) were cropped from a single photographic image of the dorsal tongue surface (Figure 4).

Each crop image was resized to 96 × 96 pixels, and then the class was predicted by applying the previous learning results. A color dot corresponding to the predicted class (blue for ‘abnormal,’ green for ‘normal,’ and red for ‘other’) was placed at the position in which the cropped image was in the original photographic image. The point mapping image was achieved by distributing color dots according to the classes on the dorsal tongue surface of the photographic image, as shown in Figure 4. With this result, the dorsal tongue surface was segmented into abnormal and normal regions. The ground truth image was also mapped by color dots using the labeled image.

**Figure 5:**
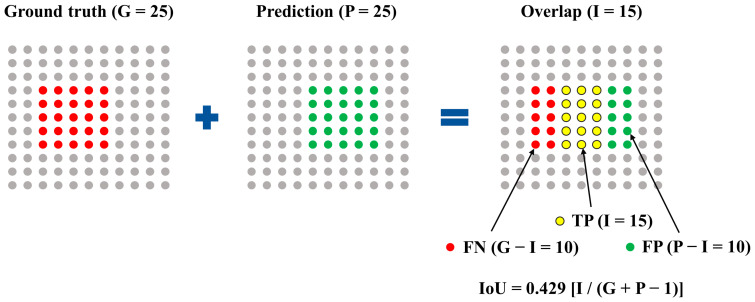
TP (true positive), FP (false positive), FN (false negative), and IoU (intersection over union) are calculated from point mapping segmentation.

This point mapping segmentation was evaluated similarly to the metric applied to DL-applied segmentation or object detection. The number of pixels corresponding to the ground truth, the prediction, and the intersection of the ground truth and prediction was defined as G, P, and I, respectively (Figure 5). The number of union pixels indicated by the ground truth and the prediction was S = G + P − I. The number of true positives, false negatives, and intersections over union (IoU) was calculated as P − I, G − I, and I/S, respectively. We calculated the precision and recall as TP/(TP + FP) and TP/(TP + FN) for each predicted point mapping image.

The average precision (AP) value was calculated by introducing PASCAL VOC evaluation metrics [27]. Abnormal, normal, and other regions were predicted on the point-mapping images of 80 test photos. The confidence value for each class was obtained by calculating the average values of the softmax predicted for the class in the prediction process using the VGG16 model for each test image. Each predicted class of point mapping region was sorted from high to low according to the confidence value. If the IoU value is 0.5 or more for each class, the prediction is correct; if not, it is false. Precision and recall values were calculated and plotted from these results to obtain AP values.

## 3. Results

The results predicted as ‘abnormal,’ ‘normal,’ and ‘other’ using the VGG16, ResNet-50, and Xeption DL algorithms were expressed as a confusion matrix, as shown in Table 3. All three DL models showed a high accuracy of 0.97. Among these models, the following analysis was performed by selecting the VGG16 model with the highest abnormal classification accuracy.

The VGG16 model calculated the precision, recall, F1-score, and AUC values for each class (Table 4). In the confusion matrix, 23 of the normal images were incorrectly predicted as abnormal, whereas only 5 of the abnormal images were incorrectly predicted as normal. Therefore, the abnormal class had a higher precision value than the normal class because the number of FP incorrectly predicted as normal or as other classes was small. On the other hand, the normal class had a higher recall value. Overall, the F1-score and accuracy values of the abnormal class evaluated were higher than those of the normal class.

**Figure 6:**
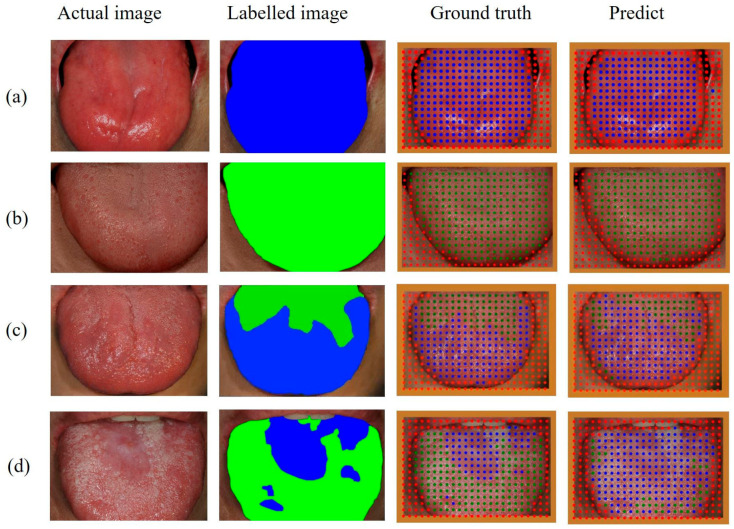
Examples of point mapping of abnormal (blue), normal (green), and other (red) regions on the photographic images of the dorsal tongue surface according to the results predicted by the VGG16 model. (**a**) Atrophic glossitis; (**b**) A normal healthy tongue; (**c**) Geographic tongue; (**d**) Oral lichen planus.

Figure 6 shows examples of point mapping to the photographs of the dorsal tongue surface. In this figure, by counting the number of points in the ground truth and the predicted images, TP, FP, and IoU values were obtained in the same way as in Figure 5, and precision, recall, and F1-score were calculated. After calculating each metric for all test images, the average precision, recall, F1-score, and IoU for each class are shown in Table 5. In addition, the threshold value of IoU was set at 0.5, and then the AP value of each class was calculated. The precision, recall, and IoU values of the abnormal class were higher than those of the normal class. In addition, the threshold value of IoU was set to 0.5, and then the AP value of each class was calculated at 0.842 and 0.706 for abnormal and normal classes, respectively.

Table 6 compares the judgments of a man (the oral medicine specialist) and the DL on normal or abnormal conditions of the local areas of the dorsal tongue surface images.

## 4. Discussion

The process by which a DL algorithm categorizes local areas on the dorsal tongue surface as normal or abnormal is similar to how a doctor observes a patient’s tongue and judges local areas as normal or abnormal on clinical examination. The AI model trained by DL according to the method of this study distinguished the normal and abnormal areas of the dorsal tongue images very accurately. The DL algorithm judged the normal uniform pattern of the filiform papillae as normal in most cases. Furthermore, the DL algorithm precisely recognized pathologically important lesions or changes, such as loss of tongue papillae, erosion, ulcers, and thick keratotic lesions. The DL algorithm made accurate judgments even when two or more abnormal features were mixed on the dorsal tongue surface: for example, a geographic tongue with partial hairy regions and a fissured tongue with atrophic or hypertrophic papillae.

Other studies that applied DL methods similar to this one reported results for oral diseases that occurred in various oral mucosal sites in the oral cavity. Heo et al. [20] showed that the diagnostic accuracy of tongue cancer was 84.7% in endoscopic images using the DensNet169 DL classification model. Yuan et al. [21] reported that the AUC values reached 0.83 to 0.88 in an independent external validation using the tongue image-based diagnostic models (APINet, TransFG, and DeepLabV3+) for gastric cancer diagnosis. Tanriver et al. [23] demonstrated that semantic segmentation of oral lesions with the EfficientNet-b7 model achieved a Dice score of 0.926 and segmentation masking using Mask R-CNN had an AP50 value of 0.762 for benign lesions, oral potentially malignant disorders (OPMD), and carcinoma in 684 oral mucosal images. Warin et al. [22] reported that the classification model had high efficiency in diagnosing OPMD with an AUC of 0.95, and Faster R-CNN showed detection performance with an AUC of 0.74 for the diagnosis of OPMD in photographic images.

However, there were some differences between the judgments of a man and the DL algorithm. The DL algorithm erroneously judged areas with high numbers of healthy fungiform papillae, like the apex and lateral borders of the tongue, as abnormal. Fungiform papillae are morphologically distinct from filiform papillae due to their larger size and red color, and they are present in high density on the tongue apex and lateral borders. In addition, shallow grooves that could be considered a normal variation were determined to be abnormal. The midline groove, a normal structure of the dorsal tongue surface, was likewise recognized as abnormal.

The disagreement between human and AI decisions may have been due to the following reasons: First, the DL algorithm misjudged the areas of the apex and lateral borders of the tongue with dense fungiform papillae as abnormal, probably due to insufficient training for the DL algorithm to learn healthy fungiform papillae. Because the crop images of the boundary regions of the tongue were excluded during the training phase of DL, the DL algorithm may not have adequately learned the normal papillary pattern in which the fungiform papillae are unevenly distributed on the dorsal tongue surface. Second, there were areas in which it was difficult to accurately distinguish between normal and abnormal tongue surfaces, even when evaluated by an expert in oral medicine. Such cases could result in considerable differences between human and AI judgments. Similarly, the transition areas where normal and abnormal features were gradually converted could have resulted in different decisions between a man and the AI because it was not easy to determine the border clearly.

The point mapping method used in this study was valuable for distinguishing and displaying normal and abnormal areas in dorsal tongue surface images. In general, when segmentation is performed on an object using a DL method such as semantic segmentation [28,29] or the U-Net [30], there are specific criteria that can explain the characteristics of the object. However, since abnormal features of lingual papillae could be randomly and locally distributed on the dorsal tongue surface, it is very difficult to label them in a standardized shape. Therefore, in this study, after extracting the local parts of the dorsal surface of the tongue, a classification was predicted for each point. As shown in Figure 6, when abnormal or normal regions were predominantly distributed on the dorsal tongue surface, this method predicted well with very good accuracy. The abnormal region was slightly overpredicted when the abnormal regions were irregularly distributed. However, overall, the segmentation was performed similarly to ground truth. Therefore, this method could effectively perform segmentation when a specific region shows an irregular distribution.

When taking clinical photographs of the dorsal surface of the tongue, several factors might have degraded the quality of the images or made those images unsuitable for DL procedures. Flashlight reflections and saliva bubbles were the typical artifacts of the clinical photographs taken. Excessive salivation often causes saliva bubbles on the dorsal surface of the tongue. Furthermore, excessive saliva on the surface of the tongue resulted in widespread flashlight reflections. Motion blur occurred when the tongue moved rapidly at the time of the camera shot. It was frequently found in the photographs taken of patients who had difficulty holding the tongue in a protruded position for an extended time. Partial blurring in the photographs was observed in the areas out of focus, mostly the lateral or posterior regions of the tongue dorsum. Dark images were obtained when the amount of light was not sufficient, even with a flashlight, and the camera lens was set with an excessively low aperture value. However, the brightness of such images could be adjusted to some extent using a graphics editing program. Original clinical photographs with severe artifacts or poor quality were filtered out and not used in this study.

This study has several limitations. First, only one expert participated in the labeling of crop images and point mapping of original photographs for VGG16 training. Therefore, there may be bias in judging the condition of the tongue mucosa. Second, crop images of the tip and lateral border of the tongue were excluded from the training of DL. Therefore, these regions with a high density of normal fungiform papillae were not judged as normal. Third, it should be noted that recognizing abnormal areas of the dorsal tongue surface using AI does not diagnose a disease. For most diseases and conditions, information from diagnostic tests such as histopathology and laboratory tests, along with the medical history, is required for a definitive diagnosis.

Follow-up studies are needed to distinguish and recognize individual abnormalities or pathological types on the dorsal surface of the tongue. Furthermore, DL methods should be able to recognize and distinguish abnormal or pathological types that appear in various regions of the oral mucosa, and the type of abnormality should be specifically determined.

## 5. Conclusions

This study analyzed the morphological features of the dorsal tongue surface using DL methods. First, the deep learning algorithm of this study can recognize and determine abnormal areas on the dorsal tongue surface with great accuracy, which may assist clinicians in evaluating diseases or conditions affecting the tongue mucosa. Second, the point mapping method used in this study could effectively perform segmentation when specific regions show an irregular distribution, such as the dorsal tongue surface, with a mixture of normal and abnormal features.

Future studies should address the limitations by involving multiple experts in the labeling process and integrating images of the tip and lateral borders of the tongue. Additionally, the research should focus on distinguishing and recognizing individual abnormalities or pathological types on the dorsal surface of the tongue, as well as expanding the scope to include various regions of the oral mucosa for accurate identification.

## Figures and Tables

**Table 1:** The criteria for abnormal or pathological changes in crop images.

Type of Change	Description
Tongue coating	accumulation of foreign substances on the tongue surface
Hairy tongue	hair-like growth of the filiform papillae
Fissures	deep furrows between tongue papillae
Papillary atrophy	a smooth, reddish tongue surface with no detailed structure of the papillae
Erosion	a pale-whitish lesion with loss of the epithelial layer only
Ulcer	a concave yellowish-white lesion with loss of the epithelial layer and part of the lamina propria
Lichenoid change	whitish epithelial thickening resembling the reticular form of oral lichen planus
Hyperkeratotic change	whitish epithelial thickening seen in lesions such as oral hyperkeratosis
Papillary hypertrophy	enlargement of the tongue papillae
Artifacts	saliva bubbles or camera flashlight reflections

**Table 2:** The number of datasets classified as abnormal, normal, or other, and the number of datasets for training, validation, and testing.

Class	Total (100%)	Training (68.8%)	Validation (15.4%)	Testing (15.8%)
Abnormal	3220	2254	483	483
Normal	2813	1881	449	483
Other	1749	1223	263	263
Total	7782	5358	1195	1229

**Table 3:** Confusion matrix obtained from the classification of the crop image dataset using the VGG16, ResNet-50, and Xeption deep learning models.

			Predict		
			Abnormal	Normal	Other
Actual	VGG16	Abnormal	476	5	2
		Normal	23	458	1
		Other	10	1	252
	ResNet-50	Abnormal	473	6	4
		Normal	15	463	4
		Other	7	2	254
	Xeption	Abnormal	462	13	8
		Normal	8	473	1
		Other	9	2	252

**Table 4:** The precision, recall, F1-score, and area under the ROC curve (AUC) values for each class.

Group	Precision	Recall	F1-Score	Accuracy	AUC
Abnormal	0.935	0.986	0.960	0.967	0.996
Normal	0.987	0.950	0.968	0.976	0.998
Other	0.988	0.958	0.973	0.989	0.998

**Table 5:** The average precision, recall, F1-score, intersection of union (IoU), and average precision (AP) values for each class were calculated from point mapping images.

Class	Precision	Recall	F1-Score	IoU	AP
Abnormal	0.717	0.737	0.727	0.695	0.842
Normal	0.650	0.641	0.645	0.590	0.706
Other	0.851	0.975	0.909	0.831	1.000
Mean value	0.739	0.784	0.760	0.705	0.849

**Table 6:** Comparison of human and artificial intelligence (AI) judgments on normal or abnormal conditions of the dorsal tongue surface images.

		AI Judgment	
		Normal	Abnormal
Human judgment	Normal	A normal uniform pattern of tongue papillae	A shallow groove that looks like a fissure (mostly the midline groove on the tongue dorsum). Areas with few filiform but many fungiform papillae (mainly at the tongue apex and lateral borders) Slightly enlarged fungiform papillae (mainly at the tongue apex and lateral borders)
	Abnormal	Mildly hairy tongue Moderate tongue coating Areas with light-toned atrophic and hyperkeratotic filiform papillae	Deep fissures Smooth and reddish areas due to atrophy of tongue papillae Thick tongue coating Erosive and ulcerous lesions Thick whitish keratotic lesions

## Data Availability

Data sharing is not applicable.
